# Implementation of Multicolor Melt Curve Analysis for High-Risk Human Papilloma Virus Detection in Low- and Middle-Income Countries: A Pilot Study for Expanded Cervical Cancer Screening in Honduras

**DOI:** 10.1200/JGO.17.00035

**Published:** 2017-08-28

**Authors:** Scott A. Turner, Sophie J. Deharvengt, Kathleen Doyle Lyons, Jorge Arturo Plata Espinal, Ethan P.M. LaRochelle, Suyapa Bejarano, Linda Kennedy, Gregory J. Tsongalis

**Affiliations:** **Scott A. Turner**, **Sophie J. Deharvengt**, **Kathleen Doyle Lyons**, **Linda Kennedy**, and **Gregory J. Tsongalis**, Dartmouth-Hitchcock Medical Center, Lebanon; **Ethan P.M. LaRochelle**, Dartmouth College, Hanover, NH; **Jorge Arturo Plata Espinal** and **Suyapa Bejarano**, La Liga Contra el Cancer, San Pedro Sula, Honduras.

## Abstract

**Purpose:**

Cervical cancer is a leading cause of cancer-related mortality in low- and middle-income countries (LMICs) and screening in LMICs is extremely limited. We aimed to implement on-site high-risk human papillomavirus (hrHPV) DNA testing in cohorts of women from an urban factory and from a rural village.

**Methods:**

A total of 802 women were recruited for this study in partnership with La Liga Contra el Cancer through the establishment of women’s health resource fairs at two locations in Honduras: a textile factory (n = 401) in the city of San Pedro Sula and the rural village of El Rosario (n = 401) in Yoro. Participants received a routine cervical examination during which three sterile cytobrushes were used to collect cervical samples for testing. hrHPV genotyping was performed using a hrHPV genotyping assay and a real-time polymerase chain reaction instrument.

**Results:**

hrHPV status across all participants at both sites was 13% hrHPV positive and 67% hrHPV negative. When hrHPV status was compared across all three testing sites, hrHPV-positive rates were approximately equal among the factory (13%), village (12%), and confirmatory testing at Dartmouth-Hitchcock Medical Center (Lebanon, NH; 14%). hrHPV genotype was compared across sites, with HPV16 showing the highest infection rate (15%), followed by HPV59 (12%), and HPV68 (11%). There was a low prevalence of HPV18 observed in both populations compared with the hrHPV-positive population in the United States.

**Conclusion:**

In collaboration with oncologists and pathologists from La Liga Contra el Cancer, we were able to provide a continuum of care once health-fair testing was performed. We established a method and implementation plan for hrHPV testing that is sustainable in LMICs.

## INTRODUCTION

Cervical cancer represents the second most commonly diagnosed cancer and the third leading cause of cancer death in women from low- to middle-income countries (LMICs). Incidence and mortality rates are five and three times greater, respectively, than in developed countries.^1,2^ Discrepancies in cervical cancer rates directly reflect low access to effective cervical cancer screening programs, physicians for follow-up care, and rapid screening technologies to segment high-risk populations.^3^

Many LMICs lack a comprehensive national cervical cancer screening program due to significant socioeconomic factors that prohibit expansion of screening programs.^4,5^ In 2014, the World Bank estimated that LMICs spent on average $200 USD per capita on health care per year compared with worldwide averages > $1,000 USD per capita. The cost of current cervical screening methods, including cytologic and molecular cotesting, are prohibitively expensive for public health applications in LMICs.^6,7^ It has been estimated that, globally, 75 LMICs have fewer than 2.5 health-care workers per 1,000 people, which fails to reach the minimum number needed to deliver basic health-care services.^8,9^ In addition, accurate screening results may take days to weeks to complete, by which time many of these patients can be lost to follow-up care.^3^

Current strategies for cervical cancer screening have been shown to dramatically reduce the incidence and mortality rate of disease.^10^ Screening programs should identify, monitor, and treat those participants at highest risk of progressing to cervical cancer. Screening recommendations have included cytology-based Papanicolaou smear (Pap) testing for women > 21 years old along with additional DNA-based high-risk human papilloma virus (hrHPV) cotesting in women > 30 years old. Recent studies have demonstrated the efficacy of forgoing cytology-based testing and using hrHPV DNA status as a primary screen. An estimated 95% to 100% of cervical cancers are the result of infection by one of 14 hrHPV types.^4,10-12^ Women negative for hrHPV have been shown to be at low risk of developing higher grade cervical intra-epithelial neoplasia (CIN) and cervical cancer for up to 5 years.^13-15^ These results have led ASCO to recommend hrHPV molecular testing as a primary screen. hrHPV testing has been shown to be more cost-effective than traditional screening by Pap and offers the potential for expanding cervical cancer screening in LMICs by substituting device-based testing for traditional cytology that requires trained cytologists, who are scarce in LMICs.^7^

Multicolor melt curve analysis (MMCA) for hrHPV detection is a polymerase chain reaction (PCR)-based DNA amplification method that enables simultaneous identification of amplified target DNA using melting temperatures of double-stranded targets from different hrHPV types. This study describes efforts to implement an hrHPV screening program including follow-up care for women at higher risk of developing cervical cancer in the LMIC of Honduras.

## METHODS

### Recruitment of Study Participants

A total of 804 women were recruited in partnership with La Liga Contra el Cancer through the establishment of women’s health resource fairs at two locations in the LMIC of Honduras: a textile factory (n = 403) in the city of San Pedro Sula, and the rural village of El Rosario (n = 401) in Yoro. Confirmatory testing was performed at the Dartmouth-Hitchcock Medical Center (DHMC). All participants were counseled regarding the use of hrHPV testing in the screening and prevention of cervical cancer and offered a gynecologic examination and primary hrHPV screening. Two participants refused hrHPV testing after counseling and were excluded from this study. Both clinics were performed during April 2016. The study protocol was reviewed and approved by the Committee for the Protection of Human Subjects at Dartmouth College (approval no. 28784) and the internal review board of the Universidad Catolica de Honduras.

### Patient Education

Several months before offering the health-care resource fairs, educational materials were developed and disseminated across the populations of potential participants. This material contained both written and diagramed information about (1) the number of people diagnosed with cervical cancer in Central America, (2) the risk factors for and symptoms of cervical cancer, (3) strategies to prevent cervical cancer, and (4) the role of early detection in reducing the risk of developing cervical cancer. Medical students and residents from the Universidad Catolica de Honduras circulated material throughout the participant populations, promoting additional verbal communication with family members and friends.

### hrHPV Sample Collection

**Fig 1:**
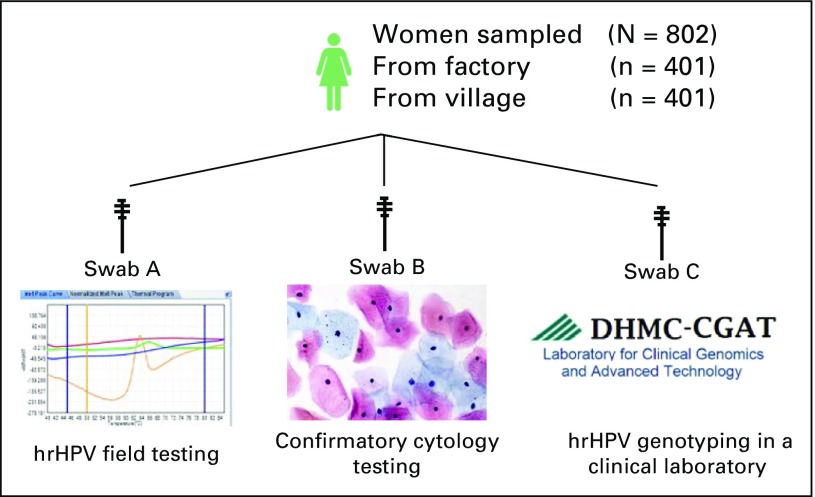
Schematic diagram showing high-risk human papillomavirus (hrHPV) testing algorithm used in this study.

Field clinics were established in repurposed areas of both the textile factory and a small village clinic. Participants were given a routine cervical examination by trained medical students and residents. During the examination, three sterile cytobrushes (Medical Packaging, Camillo, CA) were labeled with patient identifiers and used for cervical sampling. “Swab A” was put back in the packaging and used for determination of hrHPV genotype in field laboratory testing as described later in Methods. “Swab B” was immediately smeared onto a slide and fixed with 95% ethanol for microscopic examination. “Swab C” was immediately fixed with 95% ethanol, allowed to air dry, and placed back into packaging for shipping to the Laboratory of Clinical Genomics and Advanced Technologies at the DHMC in Lebanon, NH, for further evaluation (Fig 1) and confirmatory testing. Due to a reagent shortage, 35% (140 of 401) of swab A samples from the village clinic were unable to be tested on site and, therefore, were treated similarly to swab C samples and sent to DHMC for initial hrHPV testing.

### hrHPV Detection

**Fig 2:**
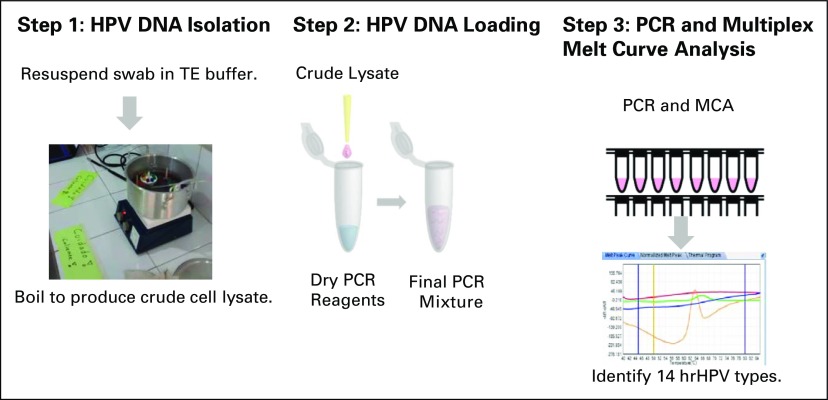
Schematic diagram of human papillomavirus (hrHPV) assay workflow. MCA, melting curve analysis; PCR, polymerase chain reaction; TE, Tris-EDTA.

A crude lysate from swab A was obtained by submerging the flocked tip of the cytobrush into a clean microcentrifuge tube containing 200 μL of 1× Tris-EDTA buffer solution (pH 8.0; Sigma-Aldrich, St. Louis, MO) and boiled (> 95°C) for 8 minutes. The crude lysate was pipetted directly into tubes containing lyophilized reagents from the MeltPro High Risk HPV Genotyping assay (QuanDx, San Jose, CA). hrHPV detection by melt curve analysis was performed according to the manufacturer’s instructions. Interpretation and reporting of the data in the field laboratories were limited to “hrHPV positive” or “hrHPV negative” using the ZSLAN software package, version 8.2.2 (Fig 2). Initial hrHPV status for 140 original samples and all samples found to be invalid in the field were tested in the clinical laboratory at DHMC. These swabs were treated similarly to field tested samples except crude lysate was obtained using 0.1M NaOH, boiled for 8 minutes and quenched with 1 M Tris-HCl buffer (Sigma-Aldrich).

### hrHPV Genotyping

The MeltPro High Risk HPV Genotyping assay uses MMCA to produce unique melt curves for all 14 hrHPV types. To ensure the accuracy of genotypes in crude lysate, a validated orthogonal genotyping method using hrHPV-specific amplification primers (Integrated DNA Technologies, Coralville, IA) was used to compare sample genotyping results. Discordant genotyping calls were then reviewed to determine if alteration to melt temperature thresholds was necessary.

### Training of Health-Care Workers Performing hrHPV MMCA

Two Honduran pathologists from La Liga Contra el Cancer traveled to DHMC for 1 week of intensive laboratory training on the aforementioned methods. The pathologists had no previous molecular training and the comprehensive training program was designed accordingly to include review of concepts of clinical molecular testing, basic infectious-disease safety training, basic molecular technique training applicable to hrHPV MMCA assay setup, and review and interpretation of data.

## RESULTS

### Cervical Cancer Risk Factors Within the Study Population:

The open study design made hrHPV screening available to all women over age 18 years, regardless of risk status. Current screening recommendations in the United States suggest women should be screened for cervical cancer (and CIN) between the ages of 21 and 65 years. The study population had average age of 40.3 years. Obesity (body mass index > 30 kg/m^2^) and multiparity status have been indicated as a risk factors in the development of cervical cancer.^16-19^ The average body mass index of the study population was 27.8 kg/m^2^. Study participants had an average of 3.4 births across both populations, with the rural village population having on average 4.7 births. Additionally, socioeconomic status and screening history have also been linked to risk of cervical cancer. In LMICs, years of education as a surrogate to socioeconomic status have been shown to be a risk factor for cervical cancer but not HPV infection rate.^20^ Although cervical cancer screening is low throughout Honduras and LMICs, some of the participants had access to previous cervical examinations in the factory (8%) and village (20%). This bias is likely because the populations this study had access to were those who had access to expanding health-care resources as part of ongoing efforts to improve health in Honduras. Demographic data of the study participants, by location, are listed in Table 1.

**Table 1:**
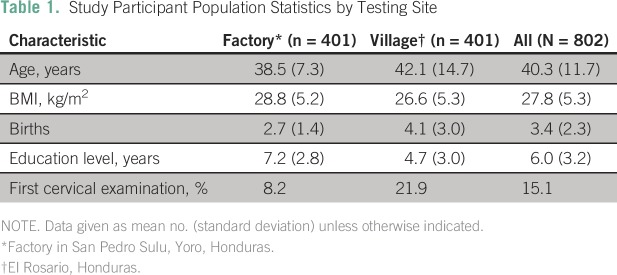
– Study Participant Population Statistics by Testing Site

### Determining hrHPV Infection Status

**Fig 3:**
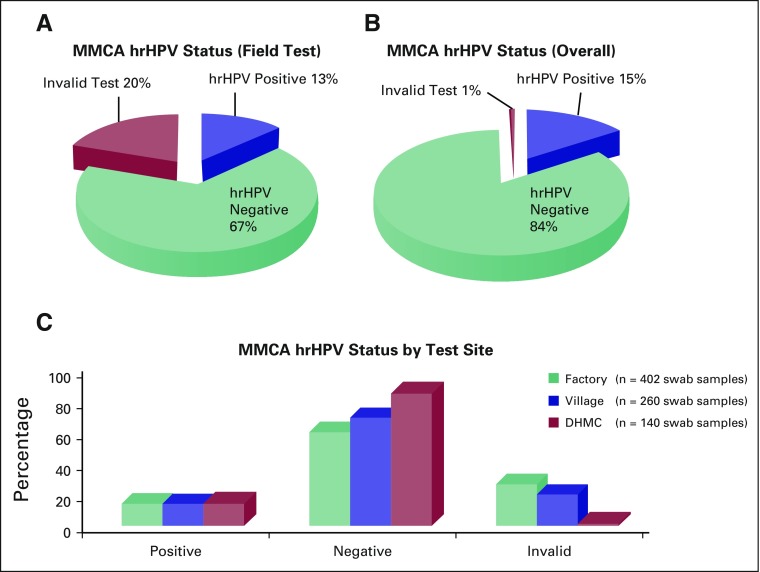
High-risk human papillomavirus (hrHPV) testing results for (A) field testing performed in Honduras, (B) overall testing including samples tested at Dartmouth-Hitchcock Medical Center, and (C) across all three test sites. MMCA, multicolor melting curve analysis.

hrHPV status across both sites was 13% hrHPV positive and 67% hrHPV negative, with 20% of samples resulting in an invalid test (Fig 3A). An invalid test was the result of a sample failing amplification for the internal β-actin PCR control or detection failure of the MMCA assay positive (ie, mix of hrHPV types) or negative (ie, known hrHPV negative) controls. All participants with an invalid result were treated similarly to those participants with positive results and their cytology samples were analyzed at La Liga Contra el Cancer, San Pedro Sula, Honduras. hrHPV status was relayed to the participants within a few days of sampling for those samples tested on site at the factory and within 2 weeks in the village, and arrangements for follow-up care at La Liga Contra el Cancer were established.

All samples with an invalid result were retested in the clinical laboratory at DHMC to more accurately determine infection rates across the study population. This resulted in an adjustment of the population’s hrHPV status to 14.3% hrHPV positive and 84% hrHPV negative; 0.7% were invalid or untestable (Fig 3B). When hrHPV status was compared across all three testing sites, hrHPV-positive rates were approximately equal among the factory (13%), village (12%), and confirmatory testing at DHMC (14%). Negative rates—60%, 69%, and 85%, respectively—were skewed largely owing to the discrepancy in invalid rates among sites. The highest invalid rate (27%) was seen at the factory site, whereas the village had an invalid rate of 19%. When initial hrHPV testing was completed in the DHMC clinical laboratory, the invalid rate dropped to 1% (Fig 3C).

### hrHPV Genotyping

**Fig 4:**
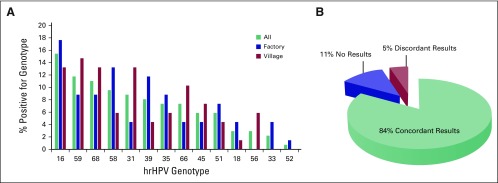
High-risk human papillomavirus (hrHPV) types detected (A) between sites and (B) concordance of results for testing between sites.

hrHPV genotype was determined and compared across sites (Fig 4A). HPV16 had the highest infection rate among all participants at 15%, followed by HPV59 (12%), HPV68 (11%), HPV58 (10%), HPV31 (9%), HPV39 (8%), HPV35 (7%), HPV66 (7%), HPV45 (6%), HPV51 (6%), HPV18 (3%), HPV56 (3%), HPV33 (2%), and HPV52 (1%). Among hrHPV-positive participants, 17% (22 of 126) were infected by multiple strains of hrHPV. HPV16 was the most prevalent in the factory population at 18% and was similar in prevalence to HPV68. HPV31 was also a common hrHPV type in the village population, with a prevalence of 13%. The most common hrHPV type in the village population was HPV59, at 15% prevalence. Interestingly, HPV16 had the highest prevalence, which is similar to that occurring in the United States; however, a low prevalence of HPV18 was observed in both populations compared with the hrHPV-positive population in the United States.

The genotypes were compared with a validated orthogonal genotyping method using hrHPV specific primers for amplification. A concordance rate of 84% (341 of 404) was observed between methods. Samples with low quality or invalid results (11%) were unable to be included in the concordance testing. Genotype miscalls occurred in up to 5% of the cases (Fig 4B).

Compared with orthogonal methods performed in the DHMC clinical laboratory, sensitivity and specificity of samples MMCA field tested for hrHPV status were 99.3% and 93.3%, respectively. Genotyping accuracy by MMCA was calculated for the six most common hrHPV genotypes found in this population, which contained at least 10 samples each. Upon initial analysis, genotyping for HPV16, HPV31, and HPV39 was 100% accurate for each genotype, whereas genotyping for HPV59, HPV68, and HPV58 was 78%, 88%, and 91% accurate, respectively, when compared with an orthogonal genotyping method. Upon review it was noted that the melt curve temperatures of a number of these samples fell just outside the indicated range set up by the manufacturer on purified DNA samples. By adjusting the melt curve temperature ranges used by the MMCA to calculate genotype, accuracy for HPV68 and HPV58 was 100%, whereas that for HPV59 remained at 78%. Genotyping accuracy in participants with multiple infections was originally found to be 56%, but, when adjusted, this accuracy rose to 80% (Table 2).

**Table 2:**
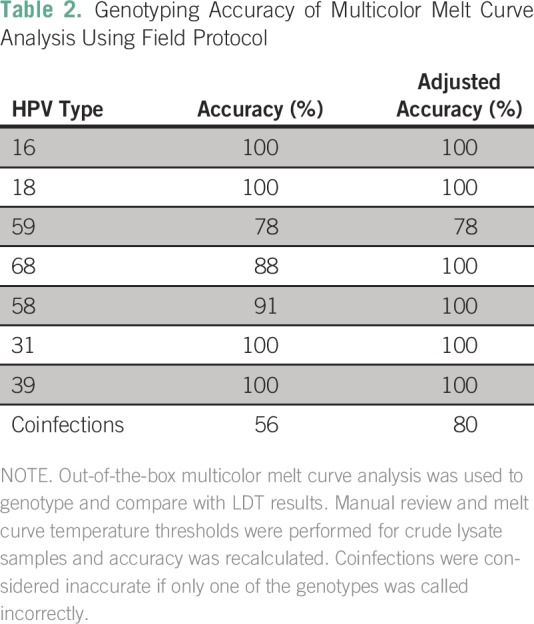
– Genotyping Accuracy of Multicolor Melt Curve Analysis Using Field Protocol

## DISCUSSION

Near-patient hrHPV testing in LMICs has the potential to improve outcomes of cervical cancer screening programs by reducing screening costs with use of high-throughput PCR devices, improving care efficiency, and improving retention of at-risk participants. The ideal LMIC test should be sensitive, specific, user friendly, and robust; require minimal equipment; and be rapid and affordable.^21^ With those attributes in mind, hrHPV MMCA near-patient testing was implemented in two separate field locations, each providing representative challenges typical of LMICs.

The hrHPV MMCA assay was highly sensitive and specific at both field sites and accuracy of HPV genotyping enabled additional participant risk stratification, potentially reducing the impact of screening on a taxed health-care system. Since the link of cervical cancer to HPV infection was made, approximately 70% of cervical cancer cases have been linked to infection with HPV16 or HPV18.^22^ This led to recommendations that participants testing positive for HPV16/18 be immediately followed up by colposcopy to diagnose CIN or cervical cancer and initiate treatment. Testing positive for the additional 12 hrHPV types puts the participant at lower relative risk for progression of CIN to cervical cancer. Recommendations for this group are to follow up after 12 to 18 months to monitor HPV clearance, but these women do not require immediate colposcopy.^23^ If those recommendations were followed in this study, only 3% of participants (25 of 802) would have been referred for immediate medical care; the additional 12% of hrHPV-positive women would have annual monitoring and escalation of care, if warranted, whereas the remaining 85% of participants would not need additional care for up to 5 years.^13^ Although HPV16/18 are more oncogenic than other hrHPV types, the distribution of hrHPV infections across the globe varies from region to region.^1^ An added benefit to genotyping all 14 hrHPV types is the opportunity for further insight into region-specific infection rates and refinement of specific population risk assessments of cervical cancer.

One of the major challenges of performing near-patient testing in LMICs is the lack of trained laboratory scientists and the limited public infrastructure typically needed for complex molecular testing. For a test to be adaptable to LMICs, it must be able to maintain sensitivity and specificity despite these momentous challenges. One way of accomplishing this is by providing thermostable reagents and packaging them as ready-out-of-the-box tests to avoid multiple procedural steps. The hrHPV MMCA achieves this by providing lyophilized hrHPV master mix prepackaged into PCR strip tubes. Temperatures at field sites in Honduras reached> 32.2°C, with high levels of humidity and no access to refrigeration for reagent storage. After 4 days of exposure to elevated temperatures, no differences in test sensitivity or specificity were observed. In addition, minimally trained personnel needed only to pipet crude lysate directly into the lyophilized reagents and place the reaction tubes into the quantitative PCR (qPCR) thermocycler for successful analysis. As noted, invalid rates were highest during the testing at the first field site but dropped in the more infrastructure-challenged village location. This was the result of testing personnel becoming better acquainted with assay setup and troubleshooting. A limitation to hrHPV MMCA is the use of a traditional qPCR instrument in an LMIC, which is typically found in a laboratory setting. In the planning phases, we considered the potential effect of power outages and found the factory had back-up generators to ensure power. In the village, which is subject to rolling brownouts and where generators are not available, we connected with the local power company to ensure our location would not have a brownout during the study period. The instrument was transported to our study locations in the back of a pickup truck and required calibration and maintenance that did not prove to be problematic in this study. Although this study was ultimately successful, development of more robust mobile instrumentation is needed to enable reliable scale-up and portability, which is desirable to reach rural populations and maximize use of devices and trained staff.

A key to reaching the goals of reducing cost and physician burden, and increasing participant retention is the rapid turnaround of screening results. An ideal near-patient hrHPV assay would provide HPV results and allow for colposcopy, if needed, in a single clinic visit. This would require HPV results to be available within 30 to 60 minutes in a routine clinical setting and perhaps even quicker in a health care–fair setting, as in this study. The hrHPV MMCA requires a lengthy PCR amplification before melting curve analysis, resulting in a 2.5-hour time to results. Batching of participant samples was performed in this study using a 96-well plate format. Although it increased throughput, batching resulted in an extended wait time. There are other commercially available methods that can perform qPCR hrHPV testing within this hour time period; however, those tests are designed for low throughput use, such as in a clinic, which, in this setting, would have resulted in a rapidly increasing backlog of samples and increased turnaround times beyond what we experienced. Furthermore, the instrumentation used for these assays has the same limitations as any qPCR machine, and reagent stability where there is a lack of cold storage would be a major factor.

There is much room for innovation and development in hrHPV testing in LMICs. In this study, we showed that the implementation of hrHPV testing using a robust assay chemistry and qPCR instrument is feasible in LMICs. Although there were several cost, technical, and environmental challenges, we were able to overcome many of them.
